# Molecular characterization of ocular dirofilariasis: a case report of *Dirofilaria immitis* in south-eastern Iran

**DOI:** 10.1186/s12879-020-05182-5

**Published:** 2020-07-16

**Authors:** Razieh Parsa, Ali Sedighi, Iraj Sharifi, Mehdi Bamorovat, Saeid Nasibi

**Affiliations:** 1 Ophthalmologist, Clinical Research Center, Pasteur Educational Hospital, Bam University of Medical Sciences, Bam, Iran; 2 Leishmaniasis Research Center, Kerman University of Medical Sciences, Kerman, Iran; 3 Research Center for Hydatid Disease in Iran, Kerman University of Medical Sciences, Kerman, Iran

**Keywords:** *Dirofilaria immitis*, Ocular, Molecular, Iran, Bam

## Abstract

**Background:**

Dirofilariasis is a zoonotic parasitic infection transmitted from animals to humans by culicid mosquitoes. Although the disease can be caused by *Dirofilaria spp.* including *Dirofilaria immitis* and *Dirofilaria repens*, human ocular dirofilariasis due to *D. immitis* is relatively rare in the world. This study was aimed to present a case of ocular dirofilariasis caused by *D. immitis* in southeastern Iran.

**Case presentation:**

A nematode extracted from the right eye of a 69-year-old man referred with clinical symptoms including itching and redness was examined. After the morphometric analysis, *Dirofilaria* parasite was detected. Afterwards, a piece of worm body was cut and DNA was extracted and a 680-bp gene fragment amplification and nucleotide sequencing were performed. Phylogenetic analysis revealed a *D. immitis* roundworm as the causative agent of infection. The patient was treated with antibiotics and corticosteroid and followed up for 1 month.

**Conclusion:**

The present study provides the second report on ocular dirofilariasis caused by *D. immitis* isolated from a human in southeast Iran. Based on the available evidence, dirofilariasis in dogs has significantly increased in endemic areas such as Iran. Therefore, physicians should be aware of such zoonotic nematodes so as to take proper and timely action and treatment against the disease.

## Background

Dirofilariasis (heartworm disease) caused by *Dirofilaria spp*. is a zoonotic parasitic infection transmitted by species of mosquitoes (Diptera: *Culicidae*) [1]. The *Dirofilaria* genus, more than 27 species of which have been identified, belongs to the family of *Onchocercidae* [2]. Microfilariae circulate in the blood of both wild and domestic animals such as dogs and cats. After about 15 days, the larvae reach the infective stage of L3 and are then introduced into a new host. Among all *Dirofilaria* species, the disease is commonly caused by *Dirofilaria immitis* and *Dirofilaria repens,* which can create pulmonary and subcutaneous nodules/ocular, respectively [3, 4].

The life cycle of *Dirofilaria* is not completed in the human body, and microfilariae have not been observed in humans [5].

Orbital infection by parasites is not common in humans, but there are reports of different genera of parasites, in particular, *Loa Loa*, *Dirofilaria* and *Onchocercae*, showing such traits across the world [6]*.* Reports of ocular dirofilariasis (OD) due to *D. immitis* are rare, whilst *D. repens* is a typical agent found in humans. Orbital, subconjunctival, and intraocular infections are clinical forms of ocular dirofilariasis in humans [7].

Clinical signs including subconjunctival and subcutaneous and rarely breast and testicular hydrocele have been reported and documented in patients with *D. repens* [7–14].

Canines and felines and other carnivores mammals are the main reservoirs of *D. immitis*, and it has been well documented that infection of dogs can potentially increase the risk of human infection [15]. Mixed infection with *D. immitis* and *D. repens* in dogs has also been reported [4]. The prevalence of canine dirofilariasis varies between 0.24–50% in different areas of the world [15]. Iranian studies have shown the prevalence to be between 0.95% in southern regions to over 60.8% in the north of the country [8]. In Iran, *Dirofilaria spp.* was first reported in a dog in 1969. According to the available data, stray dogs are also infected with *D. immitis* at different rates. Not only dog owners, but the entire community is considered to be at risk of infection [8, 16]. Serological study results have shown that approximately 5.4% of domestic dogs in Kerman province, in southeastern Iran, are infected with *D. immitis* and this fact potentially increases the incidence of the disease in humans in this region [17].

There are various methods such as stained blood smears, modified Knott’s technique, Wylie’s filtration, or isolation of adult worms in addition to morphological and molecular methods for the diagnosis of dirofilariasis in the definitive host. Such parasitological methods are applicable for all species with blood-circulating microfilariae. Enzyme-linked immunosorbent assay (ELISA), western blot, and other immunological tests are capable of detecting antigens of adult female nematodes in the final host. In the last decade, molecular identification techniques have been developed for specific diagnosis of various species of *Dirofilaria* [18].

This study was performed to report the detection of a *D. immitis* infection in the human eye through the molecular identification method in Bam, southeast of Iran.

## Case presentation

**Fig. 1:**
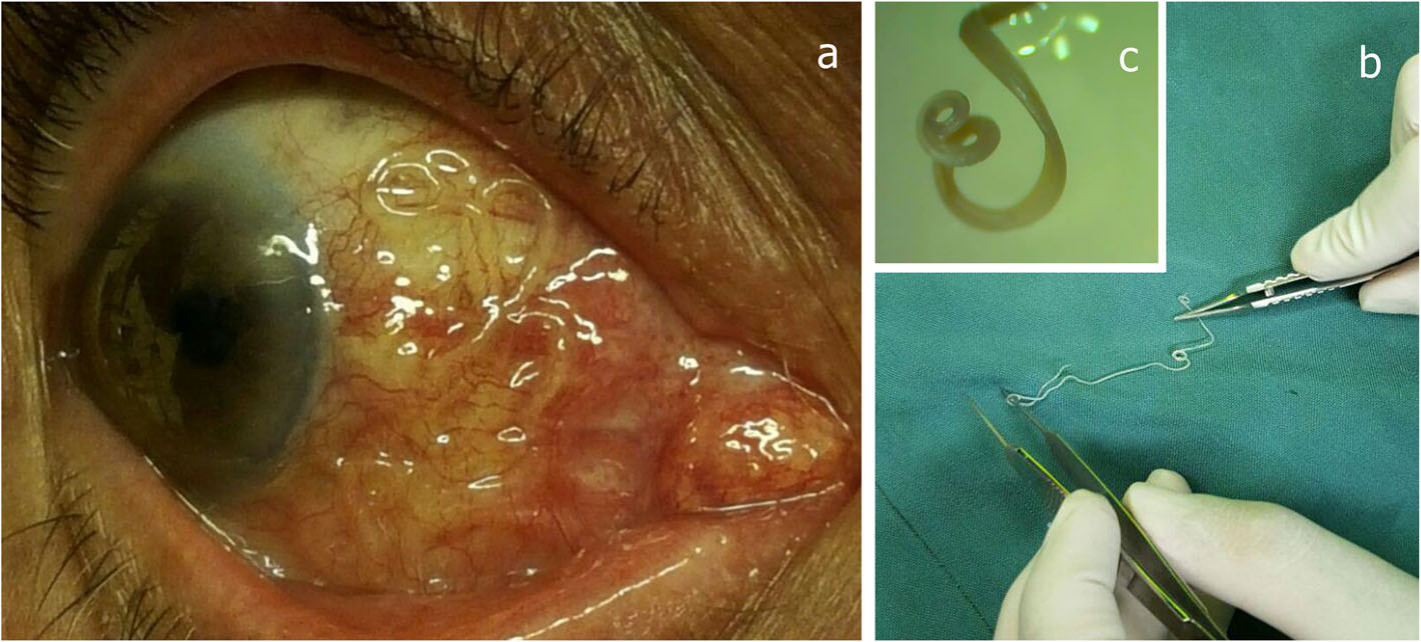
**a***Dirofilaria* roundworm in nasal sub conjunctival space in the right eye (**b**) worm after to extraction. **c***D.immitis* coiled tail

A 69-year-old man referred to Pasteur Hospital in Bam, southeastern Iran, with clinical symptoms including itching and redness of his eye. On further examination by slit lamp, a white roundworm was seen in the nasal subconjunctival space in the right eye (Fig. 1a, b, and c). Visual acuity was 7/10, IOP was normal and rest examination was unremarkable. The complete blood count (CBC) test was normal without any sign of eosinophilia. Under local anesthesia trough a conjunctival incision a white round worm was removed. After surgery, the patient was treated with antibiotics and corticosteroid and followed up for 1 month.

Morphometric analysis revealed an immature nematode 180 mm in length. After the parasite was removed, the nematode was kept in 70% ethanol for further examination. For a definite diagnosis, it was sent to the Parasitology Department of Kerman University of Medical Sciences where the *Dirofilaria* parasite was identified based on morphological keys [19].

Moreover, a piece of the worm’s body was separated, and DNA was extracted using a DNA minikit (Qiagen, Hilden, Germany). A 680-bp cox1 gene fragment was amplified using primers (Forward) 5′- CCTTTGAGTGTA-GAGGGTCAGC-3′ and (Reverse) 5′-ATTCCGCTCAAACCTCCAAT-3′ as previously described [17].

Amplification was conducted under the following cycling conditions: 94 °C for 3 min, 40 cycles of 30 s at 94 °C, 35 s at 58 °C, and 1 min at 72 °C, followed by a final extension of 7 min at 72 °C. The quality of the PCR products was assessed by gel electrophoresis.

**Fig. 2:**
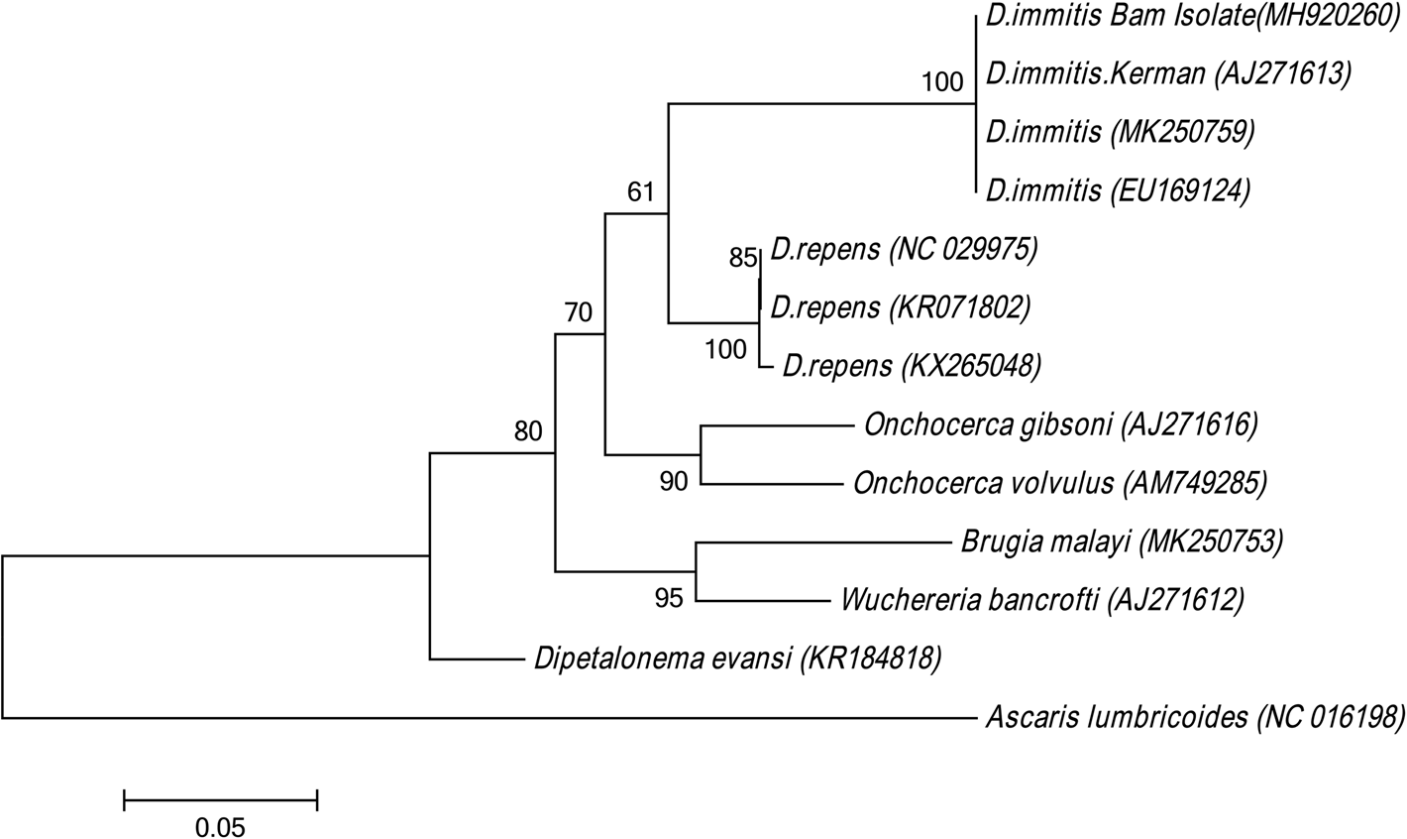
Molecular phylogenetic analysis of *Dirofilaria immitis* isolate and other Onchocercidae species based on cox1 sequences. Phylogenetic tree was inferred by Maximum Likelihood method with the highest log likelihood (− 2530.6048) based on the Kimura 2-parameter model [20]. Bootstrap values calculated from 1000 trees. The tree is drawn to scale, with branch lengths measured in the number of substitutions per site. Evolutionary analyses were conducted in MEGA6 [21]

Nucleotide sequencing was performed by the Sanger method (Macrogen Inc., South Korea). Sequencing results were aligned using BioEdit (ver.7.0.9.0) and MEGA 6.0 software and sequence identity was evaluated using the (http://www.ncbi.nlm.nih.gov/BLAST). In addition, the cox1 gene fragment was submitted to the GenBank under the accession number MH920260 as *D. immitis*. Additionally, the phylogenetic analysis produced a sister clade as compared to *D. immitis* sequences recorded in the GenBank (Fig. 2).

## Discussion and conclusion

Dirofilariasis has been considered an emerging zoonotic disease in recent years [8]. The prevalence of the disease is on the rise, which has turned it into a significant health problem in various parts of the world. Dirofilariasis is scattered throughout Iran with diverse prevalence rates in different regions of the country [8]. According to available evidence, dirofilariasis has recently increased in Iran, which is considered an endemic area. However, the reported cases appear to be far fewer than the actual cases of animal and human infection [22]. In the Old World, subcutaneous/ocular dirofilariasis cases involving *D. repens* are greater in number in comparison with pulmonary dirofilariasis cases caused by *D. immitis* [23]. The rate of *Dirofilaria* infection shows a different pattern in various geographical regions of Iran. The prevalence and spread of mosquitoes caused by the increase in temperature, availability of microfilariaemic hosts and the subsequent increase in the transmission of dirofilariasis, and specific characteristics of the parasite can increase the risk of disease in the region [24].

The disease can be diagnosed in humans using the clinical signs and parasitological, serological, histopathological, ultrasound (sonography), and molecular methods*.* In the present study, we amplified a 680-bp of cox1 gene fragment. The phylogenetic tree analysis confirmed our clinical isolate to be *D. immitis.* In recent years, several cases of human ocular dirofilariasis have been reported in Iran [7, 9, 10, 14, 25, 26]. Our literature review in this report demonstrated that most patients were diagnosed with OD through morphological methods, and only one study confirmed its results using molecular analyses [7]. In all these cases, except one, the parasite was isolated from the right eye, and ocular pain, redness, itching, tearing, and swelling of lids were the main symptoms of OD. Moreover, the anterior chamber, lateral rectus muscle, and the subconjunctival and temporal sides of the bulbar conjunctiva were the most common locations of the worm. The continued presence of worms in the eye can lead to complications including damaged vision, loss of sight, floaters, or other losses of visual acuity [23]. It is well accepted that the removal of the worm with surgery is the best approach to treating OD patients while chemotherapy is not recommended due to the lack of microfilaria in human blood. However, risks and side effects of surgical extraction of worms from the optic nerve area make this intervention extremely complicated.

To our knowledge, the present study provides the second report on ocular dirofilariasis by *D. immitis* isolated from a human in the southeast of Iran, which has favourable climatic conditions for the proliferation of the parasite. The epidemiology of parasitic ocular diseases is directly related to sanitation, environmental conditions, and the habits of the patient [26]. In this study, the patient lived in a rural area of the southern part of Bam city, which is located in a dry and semi-dry region with long hot days throughout the year. The patient had a history of exposure to domestic and stray dogs around his home.

Reducing the infection of reservoirs (stray and domestic dogs), public awareness, informing dog owners, and reducing mosquito populations can be beneficial for control and prevention of dirofilariasis. Due to the increasing number of human infection reports in recent years on the one hand, and the high population of stray dogs and the increase in the rearing and keeping domestic dogs and cats in Iran on the other, it is necessary to pay more attention to this zoonotic disease, especially in hyperendemic areas. It should be noted that in Iran, the lack of dog population management has resulted in an increase in stray dogs.

It should be taken into account that the epidemiology of parasites plays a major role in the implementation of control strategies. The most effective measure for preventing zoonotic diseases in dogs and cats is anthelmintic treatment, where prophylactic doses of ivermectin or milbemycin are given to animals regularly in the framework of the program prepared by the World Health Organization [27, 28].

In conclusion, *D. immitis* could cause ocular dirofilariasis in human in areas where the climate conditions are favorable for disease propagation. The present study provides the second report on ocular dirofilariasis by *D. immitis* isolated from a human in southeast of Iran whereas most reports in Iran have indicated OD cases transmitted by *D. repens*. This fact highlights the importance of conducting epidemiological studies using geographic information systems (GIS) and remote sensing (RS) methods for dynamic and distribution prediction of dirofilariasis in Iran. Therefore, physicians should be aware of such zoonotic nematodes so as to be able to take proper action against this disease.

## Data Availability

Patient sample (extracted worm and DNA) are deposited in the Parasitology Department of Kerman University of Medical Sciences. Nucleic acid sequences were submitted to GenBank® (NCBI) and accession numbers are included in MS. (Fig. 2).

## Abbreviations

OD: Ocular dirofilariasis
DNA: Deoxyribonucleic Acid
ELISA: Enzyme-linked immunosorbent assay
PCR: Polymerase chain reaction
bp: Base pair
GIS: Geographic information systems
RS: Remote sensing
